# Factors Affecting Regional Per-Capita Carbon Emissions in China Based on an LMDI Factor Decomposition Model

**DOI:** 10.1371/journal.pone.0080888

**Published:** 2013-12-06

**Authors:** Feng Dong, Ruyin Long, Hong Chen, Xiaohui Li, Qingliang Yang

**Affiliations:** 1 School of Management, China University of Mining and Technology, Xuzhou, Jiangsu, China; 2 School of Foreign Languages, Yantai University, Yantai, Shandong, China; Universidad Veracruzana, Mexico

## Abstract

China is considered to be the main carbon producer in the world. The per-capita carbon emissions indicator is an important measure of the regional carbon emissions situation. This study used the LMDI factor decomposition model–panel co-integration test two-step method to analyze the factors that affect per-capita carbon emissions. The main results are as follows. (1) During 1997, Eastern China, Central China, and Western China ranked first, second, and third in the per-capita carbon emissions, while in 2009 the pecking order changed to Eastern China, Western China, and Central China. (2) According to the LMDI decomposition results, the key driver boosting the per-capita carbon emissions in the three economic regions of China between 1997 and 2009 was economic development, and the energy efficiency was much greater than the energy structure after considering their effect on restraining increased per-capita carbon emissions. (3) Based on the decomposition, the factors that affected per-capita carbon emissions in the panel co-integration test showed that Central China had the best energy structure elasticity in its regional per-capita carbon emissions. Thus, Central China was ranked first for energy efficiency elasticity, while Western China was ranked first for economic development elasticity.

## Introduction

In the 15th Conference of the Contracting Parties under the “United Nations Framework Convention on Climate Change” and the 5th Conference for the Parties under the “Kyoto Protocol” held during December 2009 in Copenhagen, Denmark, the Chinese government solemnly promised that the carbon emissions per unit GDP would be decreased by 40–45% by 2020 from the 2005 levels. According to the “Kyoto Protocol” agreed at the climate conference held in Copenhagen, there was a consensus about how to reduce greenhouse gas emissions and to keep the atmospheric temperature at a reasonable level. China is believed to be the highest carbon producer in the world, so it feels that it is imperative to transform its current status by implementing technological and systematic innovation, fundamentally abandoning its old-fashioned economic development pattern, and adopting an energy saving and low-carbon economic development path to improve its energy efficiency, thereby rationalizing people’s lifestyles and consumption patterns.

The “Tokyo Protocol” and “Copenhagen Annual Conference” declared clear and concrete constraints on all nations regarding their carbon emission reduction obligations, but these issues are still under discussion as to how to evaluate a nation or district’s carbon emissions, and which indicator should be utilized for scientific measurements. Many scholars and professionals have explored this issue in effective ways. Mielnik et al. proposed that the carbon dioxide emissions per energy unit could be used as the main evaluation criteria to address climate change and the economic development models of developing countries [1]. Ang suggests that the change in the energy consumption per unit GDP could represent the regional carbon dioxide emission situation [2]. Zhang et al. thought that up-to-date evaluation indexes such as the per-capita industrialized cumulative carbon emissions and carbon emissions per unit GDP would be more likely to adhere to scientific, fair, and reasonable principles [3]. Sun expressed the opinion that the carbon dioxide emissions per unit GDP would be a good index for comparing decarbonization among countries [4]. In addition to the above indicators, the per-capita carbon emissions indicator is an important measure of regional carbon emissions level and strength. The per-capita carbon emissions indicator appears in many study domains because many scholars believe it is a better option that reflects regional fairness rules, thereby protecting the interests of developing countries. For example, Lin and Li estimated the actual mitigation effects of the five north European countries using the difference-in-difference (DID) method [5]. Andrew et al. examined the regional and temporal differences in the statistical relationship between carbon dioxide emissions and population size [6]. Lanne et al. considered the per-capita carbon emissions trends in 16 early-developed countries for the period 1870–2028 using a multiple-break time series method [7]. Wang et al. calculated China’s per-capita carbon emissions in terms of its gravity center, as well as its variation trends, features, and explanations during 1995–2005 [8]. Tian and Zhang calculated China’s per-capita factor analysis model for carbon emissions decomposition based on the Generalized Fisher Price Index method (GFI) [9]. Rajaratnam and Kanthi developed a conditional equilibrium correction model (ECM) for quantifying the relationship between Australia’s per-capita GDP and per-capita carbon emissions [10]. Lee and Chang used Panel Seemingly Unrelated Regressions Augmented Dickey–Fuller (SURADF) unit-root tests to determine whether the stochastic convergence and β-convergence of the per-capita carbon emissions were supported in OECD countries [11]. By following the principle of regional fairness, the authors also employed per-capita carbon emissions as an indicator when measuring the carbon emissions situation of all provinces in China, which differ greatly in their economic and environmental status. In addition, Data Envelopment Analysis (DEA) has often been used to study the environmental or energy efficiency in recent years. For example, Zaim, Zofío, and Zhou evaluated the carbon dioxide emission performance of OECD countries and others regions at the macro-level using different DEA models [12]–[14]. Sebastian and Gutiérrez proposed a non-parametric frontier approach for modeling the relationships among populations, GDP, energy consumption, and carbon dioxide emissions [15]. Zhou et al. built MCPI model based on the Malmqusit index and used it to measure the carbon emission efficiency of 18 countries with the highest global carbon emissions [16]. Wang et al. set up the Malmquist index using a DEA model containing undesired outputs, which was used to study dynamic changes in the carbon emission performance in China [17].

Decomposition analysis is often used in studies related to carbon emissions. For example, Fan et al. used the Adaptive Divisia Decomposition method (AWD) to divide the factors affecting China’s carbon intensity during 1980–2003 and showed out that the primary energy intensity had a significant effect on the carbon intensity [18]. Zhang et al. analyzed the factors related to China’s total carbon emissions and carbon intensity using data from 1991 to 2006 with the complete decomposition method developed by Sun [19], which demonstrated that the energy intensity contributed most to the decline in the carbon emissions and carbon intensity [20]. The crux of decomposition analysis is the elimination of decomposed residuals. In 2008, Ang et al. introduced a Logarithmic Mean Divisia Index method (LMDI) to solve this problem. Ang compared this new method with three existing methods and summarized the respective decomposition formulae for various applications [21]. Ang later proposed the improved LMDI decomposition method, which has been used widely to analyze factors in the field that affect carbon emissions. Chunbo, Tunc, and Claudia analyzed the main factors affecting carbon emission changes in China, Turkey, and Mexico by decomposing the carbon emissions and/or the carbon emission intensity using the LMDI approach [22]–[24]. Ang discussed which was the “best” decomposition method and analyzed the consistency on the choice of decomposition methods in empirical studies about decomposition analysis for policymaking in energy [25]. The LMDI approach has also been adopted for monitoring economy-wide energy efficiency trends. For example, Ang et al. proposed accounting frameworks for tracking energy efficiency trends based on the LMDI decomposition technique, which has a number of desirable properties [26]. Dong and Wu established accounting frameworks for the energy performance index (EPI) based on the LMDI approach to analyze the energy consumption of Beijing [27]. Using the LMDI approach, Baležentis et al. analyzed the energy intensity trends in the Lithuanian economy as a whole as well as in separate economic sectors [28].

The LMDI approach is a type of index decomposition analysis (IDA), while another popular decomposition technique for energy and emissions is structural decomposition analysis (SDA). Three aggregation issues are inherent in SDA studies, namely sector aggregation, spatial aggregation and temporal aggregation [29]. Su and Ang examined new methodological developments in SDA and compared four SDA methods analytically and empirically by decomposing changes in China’s CO_2_ emissions [30]. Su et al. investigated analytically the possible effects of sector aggregation, spatial aggregation and temporal aggregation on SDA and conducted empirical studies using the data of CO_2_ emissions [31], [32], [29]. In recent years, many scholars have used the SDA approach for studying carbon emissions and reported many interesting findings and recommendations [33]–[36].

The decomposition method is a relatively common method for the analysis of regional carbon emissions change. However, many studies merely decompose the carbon emissions into a number of factors without further exploration of the influential mechanisms and dynamic changes in these factors. Based on the results of factor decomposition for per-capita carbon emissions, we selected the Panel Co-integration Analysis method and conducted an in-depth exploration of the long-term and short-term dynamic changes, and the factors with strong effects on the per-capita carbon emissions.

As mentioned above, the main indexes available for evaluating the regional carbon emissions situation include the total carbon emissions, per-capita carbon emissions, carbon emissions per unit GDP, and the carbon emissions performance based on the DEA model, while the per-capita carbon emissions indicator is very important for demonstrating the regional carbon emissions level. In summary, the LMDI decomposition approach proposed by Ang et al. is used more frequently for carbon emissions decomposition, besides the LMDI method has recently become popular in SDA studies related to energy and emissions. China’s regional carbon emissions vary greatly, i.e., there was an eightfold difference in the per-capita carbon emissions of the highest and the lowest provinces in 2009, so we used the LMDI factor decomposition model–panel co-integration test two-step method to study the main factors affecting the per-capita carbon emissions in Chinese provinces, and explore the influential mechanisms and dynamic changes in the per-capita carbon emissions, thereby facilitating a quantitative analysis of the emissions reduction strategy.

This paper is organized as follows. Section 2 presents the results of the regional per-capita carbon emissions in Chinese provinces. Section 3 describes the LMDI approach used to decompose the per-capita carbon emissions and the decomposition results in China. Based on the factors decomposed in Section 3, the panel co-integration analysis of the factors that affect the regional per-capita carbon emissions is presented in Section 4. Finally, we conclude this study.

## Calculation of Regional Per-capita Carbon Emissions

This study analyzed the energy consumption of total coal, total oil, and natural gas, which were obtained from the China Energy Statistical Yearbook published by China’s National Bureau of Statistics (CNBSa) [37], to calculate carbon emissions for each area. In this study, total coal included various types of coal resources, such as raw coal, cleaned coal, and coke. Oil included crude oil, gasoline, kerosene, diesel oil, and other petroleum products. The formula used was as follows:
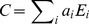
(1)Where, 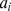 is the i type carbon emission coefficient and 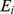 is the i-th energy consumption (standard coal). The carbon emission coefficients of the three types of energy are referred to in the research results presented by Xu, Hu et al., and an IPCC report [38]–[40]. In addition, we calculated the per-capita carbon emissions from 1997 to 2009 by dividing the total population by the total carbon emissions. Eastern China has 11 provinces, i.e., Beijing, Tianjin, Hebei, Liaoning, Shanghai, Jiangsu, Zhejiang, Fujian, Shandong, Guangdong, and Hainan. Central China has eight regions, i.e., Shanxi, Jilin, Heilongjiang, Anhui, Jiangxi, Henan, Hubei, Hunan. Western China contains Inner Mongolia, Guangxi, Chongqing, Sichuan, Guizhou, Yunnan, Shaanxi, Gansu, Ningxia, Qinghai, and Xinjiang, i.e., 11 provinces in total (there are no energy consumption data for Tibet and Taiwan). For the regions mentioned above, their regional per-capita carbon emissions are shown in Table 1.

**Table 1:** The regional per-capita carbon emissions (unit: tons per-capita) in China.

Region	1997	2009	The mean	Region	1997	2009	The mean
Beijing	5.6802	6.0726	5.9115	Hunan	1.5627	3.7365	2.2603
Tianjin	6.2658	8.9827	7.8615	Guangdong	2.3462	4.5819	3.2994
Hebei	3.6549	7.7655	5.1951	Guangxi	0.9932	2.5300	1.5772
Shanxi	9.1547	16.3462	12.4616	Hainan	0.9161	3.0314	1.8501
Inner Mongolia	5.0146	20.7720	9.9975	Chongqing	1.9780	4.6769	2.7046
Liaoning	4.9356	9.1021	6.5527	Sichuan	1.5908	3.6425	2.1210
Jilin	4.1991	7.0579	4.9820	Guizhou	2.8699	5.8755	3.9644
Heilongjiang	4.3831	7.0566	5.0307	Yunnan	1.6772	4.2044	2.5374
Shanghai	7.5084	10.2739	8.8732	Shaanxi	2.1953	5.8814	3.3198
Jiangsu	2.7551	6.4008	4.1941	Gansu	2.2219	4.0027	2.9960
Zhejiang	2.5917	6.3191	4.1964	Qinghai	2.5514	6.1666	3.7683
Anhui	1.7384	4.2912	2.6286	Ningxia	4.3157	15.9706	9.4979
Fujian	1.3519	4.7129	2.7021	Xinjiang	4.0555	8.3971	5.2403
Jiangxi	1.3141	2.6950	1.8814	Eastern China	3.1543	6.8472	4.6294
Shandong	2.6473	8.4324	4.8081	Central China	2.7278	5.7547	3.8522
Henan	1.8228	5.3546	3.2417	Western China	2.1983	5.8068	3.3659
Hubei	2.3130	4.7763	3.1608	China	2.7361	6.2079	4.0169

Note: In this study Eastern China comprises 10 provinces, Central China, eight provinces, and Western China, 11 provinces, China, 30 provinces.

Table 1 shows that in 1997, Eastern China, Central China, and Western China ranked first, second, and third in the per-capita carbon emissions, whereas in 2009, the ranking changed to Eastern China, Western China, and Central China. In addition, the per-capita carbon emissions in Western China exceeded those of Central China in 2009. In the ranking of the 13-year average per-capita carbon emissions, the top five provinces were Shanxi, Inner Mongolia, Ningxia, Shanghai, and Tianjin. Shanxi and two other provinces that ranked in the top three were affected by their large coal reserves, which led to greater energy consumption. However, this was not the case for Shanghai and Tianjin where the higher level of per-capita carbon emissions were closely related to their higher level of economic development. Finally, Guangxi, Hainan, Jiangxi, Sichuan, Hunan had comparatively low levels, which reflected their economic growth.

**Figure 1:**
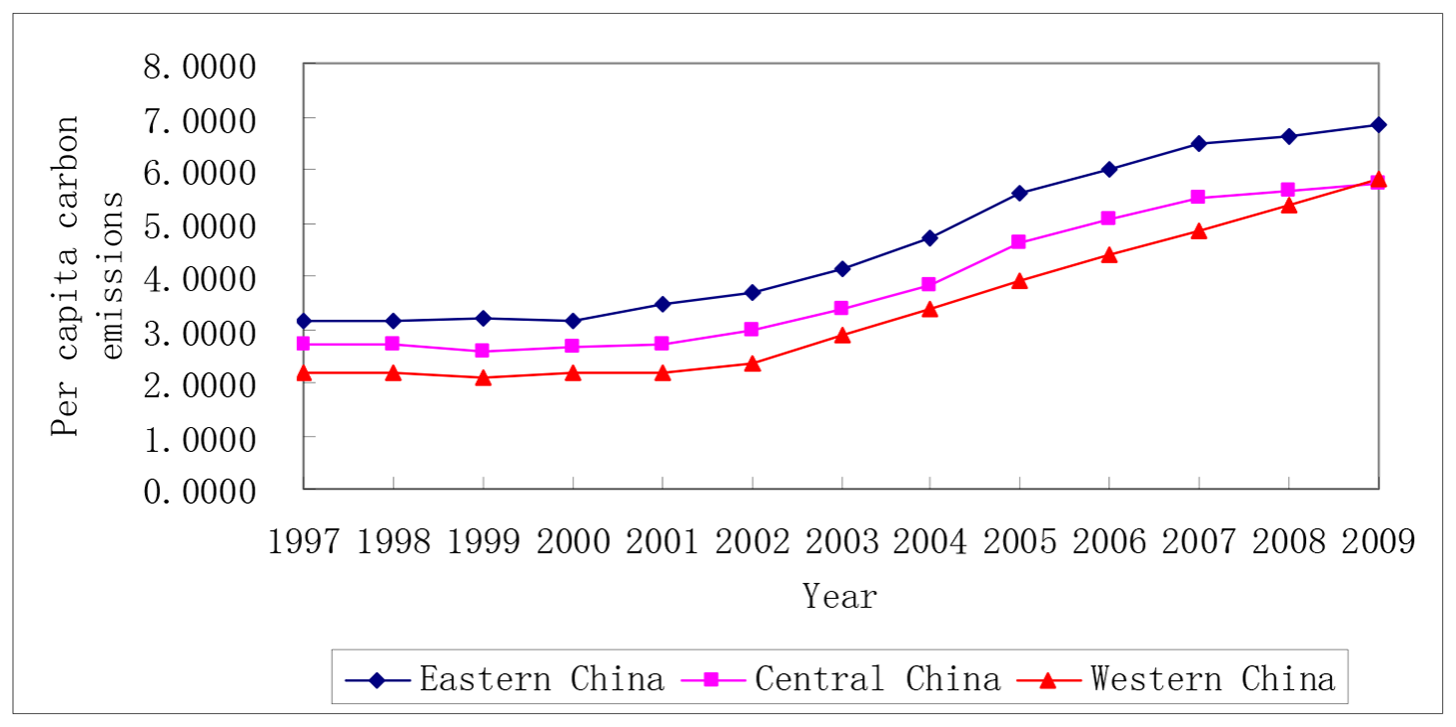
Changes in the per-capita carbon emissions in the three major economic regions of China.

Figure 1 shows that from 1997 to 2002, the three main economic regions grew slowly in terms of their per-capita carbon emissions, whereas they developed rapidly after 2002. In other words, 2002 was the turning point that corresponded to an anomalous increase in China’s energy consumption per unit GDP (2002–2005). Between 1997 and 2009, the average growth rates of the per-capita carbon emissions in Eastern China, Central China, and Western China were 6.67%, 6.42%, and 8.43%, respectively, where Western China was highest.

## LMDI Factor Decomposition of the Regional Per-capita Carbon Emissions

### 1. LMDI factor decomposition model

According to the Kaya Identity, the regional carbon emissions can be decomposed as follows:
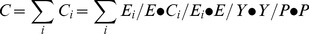
(2)


Where, 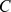 is the regional total carbon emissions, 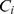 is the carbon emissions for the i-th energy, 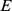 is the primary energy consumption, 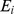 is the i-th energy consumption of standard coal, 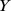
is the regional GDP, and 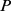 is the total regional population. Furthermore, the carbon emissions are divided by the population to obtain the per-capita carbon emissions:
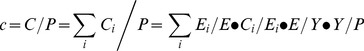
(3)


Eq. (3) shows that the regional per-capita carbon emissions are affected by four factors:

- Energy structure (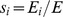), the share of the i-th primary energy relative to the total energy consumption;
- Carbon emission coefficient (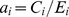), the carbon emissions of the i-th primary energy consumption;
- Energy efficiency (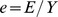), the energy consumption required to generate the GDP per unit, which was measured based on the energy intensity;
- Economic development (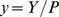), measured based on the regional per-capita GDP.

Therefore, the per-capita carbon emissions can be expressed as follows.
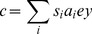
(4)


The change in the regional per-capita carbon emissions from year h to year t can be expressed as follows.
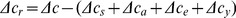
(5)


According to Eq. (5), the change in the regional per-capita carbon emissions can be decomposed into four factors:

- Energy structure factor: 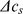;
- Carbon emission coefficient factor: 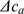;
- Energy efficiency factor: 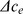;
- Economic development factor: 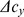.

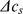
, 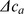, 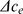, and 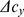 express the contributions of the changes in each factor as changes in the per-capita carbon emissions, while 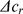 denotes the decomposition residual.

In this study, we used the LMDI decomposition method introduced by Ang et al. The decomposition factors are expressed as follows.
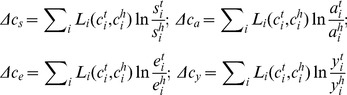
(6)


Where, 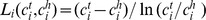


### 2. The LMDI-based decomposition result of regional per-capita carbon emissions

In Eq. (2), i represents the type of energy consumption, including total coal, total oil, and natural gas, which are all converted into standard coal. The data of the total coal, total oil, and natural gas for each region were taken from China Energy Statistical Yearbook [37]. In CESY the original data are given for 42 sectors. According to Eq. (6) the change in the per-capita carbon emissions in the provincial area from 1997 to 2009 can be decomposed into the energy structure factor, carbon emissions coefficient factor, energy efficiency factor, and economic development factor. The carbon emissions coefficients based on the IPCC statistics will remain the same during a certain period so the decomposition result for the carbon emissions coefficient is zero. In addition, the decomposition value of each factor is divided by the total variation from 1997 to 2009, so the share of each decomposition factor can be obtained. The results are shown in Table 2. The detailed decomposition results for each region and year can be seen in (Tables S1, S2, S3, S4, S5, S6, S7, S8, S9, S10, S11, S12 in Appendix S1).

**Table 2:** The LMDI-based decomposition results (1997–2009).

Region	Share of the energystructure factor	Share of the energyefficiency factor	Share of the economic development factor	Region	Share of the energystructure factor	Share of the energyefficiency factor	Share of the economic development factor
Beijing	−0.3534	−8.1311	9.4845	Hunan	−0.0041	−0.2980	1.3020
Tianjin	−0.0198	−2.2543	3.2742	Guangdong	−0.0053	−0.5168	1.5222
Hebei	0.0003	−0.4099	1.4095	Guangxi	−0.0092	−0.1147	1.1239
Shanxi	−0.0069	−1.0079	2.0148	Hainan	−0.0040	0.2491	0.7549
Inner Mongolia	−0.0038	−0.2587	1.2626	Chongqing	−0.0021	−0.5020	1.5040
Liaoning	−0.0084	−0.7521	1.7605	Sichuan	−0.0114	−0.3402	1.3516
Jilin	−0.0065	−1.3563	2.3628	Guizhou	−0.0016	−0.6791	1.6807
Heilongjiang	0.0041	−0.6905	1.6865	Yunnan	−0.0010	0.0511	0.9499
Shanghai	−0.0764	−1.3801	2.4564	Shaanxi	−0.0101	−0.4037	1.4138
Jiangsu	−0.0033	−0.4341	1.4374	Gansu	−0.0061	−0.8009	1.8070
Zhejiang	−0.0007	−0.2353	1.2360	Qinghai	−0.0276	−0.2928	1.3204
Anhui	0.0020	−0.0965	1.0945	Ningxia	0.0005	−0.0308	1.0303
Fujian	0.0082	0.2126	0.7793	Xinjiang	0.0100	−0.1746	1.1646
Jiangxi	−0.0058	−0.5021	1.5079	Eastern China	−0.0067	−0.4330	1.4397
Shandong	0.0022	−0.0563	1.0542	Central China	−0.0027	−0.4424	1.4451
Henan	0.0013	−0.1182	1.1169	Western China	−0.0050	−0.2332	1.2382
Hubei	−0.0107	−0.3646	1.3753	China	−0.0056	−0.3885	1.3940

The decomposition results show that the most powerful driver of the per-capita carbon emissions in Eastern China, Central China, Western China, and China between 1997 and 2009 was economic development. Undoubtedly, China’s economy grew rapidly between 1997 and 2009, and the per-capita carbon emissions were closely associated with economic development. Thus, the rapid development of the economy led to vigorous growth in the per-capita carbon emissions, which was exemplified by the high average annual growth in China’s per-capita carbon emissions (7.1%) between 1997 and 2009. The main effect of economic growth on the per-capita carbon emissions in Eastern China was similar in Central China, but greater than that in Western China. The inhibitory effect of energy efficiency on the increase in the per-capita carbon emissions was much greater than that of the energy structure. The inhibitory effect of energy efficiency improvement on the per-capita carbon emissions in Eastern China was similar to that in Central China, but larger than that in Western China. Furthermore, the inhibitory effect of the energy structure change on the increased per-capita carbon emissions was very weak and the shares of the energy structure factor in Eastern China, Central China, and Western China were all less than 1%. Indeed, the coal-oriented energy structure in the three economic regions remained unchanged. The proportion of coal was >70% and the only difference was a slight increase in the proportion of natural gas. The micro-adjustment of the energy structure had a limited effect on the change in the per-capita carbon emissions. For these provinces, the effect of economic growth on the per-capita carbon emissions was high in the top five provinces, i.e., Beijing, Tianjin, Shanghai, Jinlin, and Shanxi, which comprised three in Eastern China, one in Western China, and one in Central China. By contrast, the inhibitory effects of energy efficiency were highest in five other provinces: Beijing, Tianjin, Shanghai, Jinlin, and Shanxi, which comprised three in Eastern China, one in Central China, and one in Western China.

## Panel Co-integration Analysis of the Factors that Affected the Regional Per-capita Carbon Emissions

### 1. Panel co-integration analysis method

The LMDI decomposition method was used to decompose the change in the per-capita carbon emissions between 1997 and 2009 into the energy structure factor, the energy efficiency factor, and the economic development factor. We also studied how these three factors affected the per-capita carbon emissions in Eastern China, Central China, and Western China using the panel co-integration test method. The basic idea of co-integration is to verify whether there is a long-term stable combined relationship between unstable variables. If this combination is also a stationary sequence, we can conclude that these variables have achieved co-integration relationship.

#### 1.1. Panel data unit-root test

The difficulty of the unit-root test for Panel data is that we consider the heterogeneity of the cross-section, but we also construct a higher potential statistic. The Panel data unit root test method is not unified and the more commonly used methods are the LLC test, Breitung test, IPS test, Fisher ADF, Fisher PP test, and Hadri test. Of these, the LLC test, Breitung test, and Hadri test are the same-root test methods, whereas the IPS test, Fisher ADF, and Fisher PP test are different-root test methods. Moreover, the LLC test, Breitung test, IPS test, Fisher ADF, and Fisher PP test assume that the unit root exists, whereas the Hadri test assumes no unit root.

#### 1.2. Panel data co-integration test

There are two ways of performing the co-integration test with Panel data: one based on the maximum likelihood ratio, and another based on the residual. It is well known that the Johansen Fisher test focuses on the maximum likelihood ratio, whereas the Pedroni test and Kao test prefer to use the residual of the E–G two-step method. We used the Pedroni test.

The Pedroni test is applied mainly to heterogeneous panels and it has seven co-integration statistics: four are interclass statistics (Panel V, Panel Rho, Panel PP, and Panel ADF) and three are group statistics (Group Rho, Group PP, and Group ADF). Panel V, Panel Rho, Panel PP, Group Rho, Group PP Phillips, and Perron statistics use nonparametric tests, whereas Panel ADF and Group ADF use the ADF test. The null hypothesis of the seven statistical tests is no co-integration relationship whereas the alternative hypothesis of the interclass statistic requires a uniform co-integration coefficient for every cross-section unit and the group statistic allows variation.

### 2. Indicators and Data

In this study, the energy structure factor (ES) is denoted by the coal share of the total energy consumption, energy efficiency factors (energy intensity, IEC) are expressed as the energy consumption per unit GDP, and the economic development factors (RY) are represented by per-capita GDP. The data were derived from: (1) China Energy Statistical Yearbook published by China’s National Bureau of Statistics (CNBSa) [37]; and (2) China Statistical Yearbook published by China’s National Bureau of Statistics (CNBSb) [41], which covered 1997 to 2009. The annual data were all converted into 2000 constant prices using the deflator index. Finally, to ensure the stability of the variables, we took the natural logarithm of each variable, which were denoted as LRC, LES, LIEC, and LRY.

### 3. Co-integration Test Results

#### 3.1. Panel data unit-root test result

Using the six methods mentioned above for the panel data unit-root test, unit-root tests of the four variables (LRC, LES, LIEC, and LRY) are carried out for Eastern China, Central China, and Western China. Due to the article length limit, we do not list the unit-root test results for the three economic regions. The Hadri test results were not obvious and in many cases the level value, the first-order difference value, and the second-order difference value were all significant. Therefore, the Hadri test results were excluded. As stated previously by Harris and Tzavalis [42], the LLC test is unreliable for a short time span, so we only determined the variables based on the Breitung test, IPS test, Fisher ADF test, and Fisher PP test. The test results for the four variables, i.e., LRC, LES, LIEC, and LRY, were all unstable. This comprehensive analysis showed that the four variables had unit-roots in the three economic regions.

#### 3.2. Panel data co-integration test results

The unit-root test results showed that the four variables, i.e., the regional per-capita carbon emissions, energy structure, energy efficiency, and economic development, were not stable. Therefore, we tested whether the four variables had a co-integration relationship prior to the panel data regression, which would have caused spurious regressions. In this study, we tested whether there was a co-integration relationship between the four variables using the seven statistics included in the Pedroni test methods. The test results are shown in Table 3.

**Table 3:** Pedroni co-integration test results for LRC, LES, LIEC, and LRY.

Statistics	Panel V	Panel Rho	Panel PP	Panel ADF	Group Rho	Group PP	Group ADF
Eastern China	−1.436201 (0.9245)	0.448341 (0.6730)	−12.32185^a^ (0.0000)	−5.958716^a^ (0.0000)	2.321724 (0.9899)	−7.967557^a^ (0.0000)	−3.468141^a^ (0.0003)
Central China	−3.290785 (0.9995)	2.185328 (0.9856)	−10.42988^a^ (0.0000)	−2.585709^a^ (0.0049)	2.965013 (0.9985)	−13.74530^a^ (0.0000)	−2.191878^b^ (0.0142)
Western China	−1.671686 (0.9527)	0.589782 (0.7223)	−9.058999^a^ (0.0000)	−2.495073^a^ (0.0063)	1.874075 (0.9695)	−16.11765^a^ (0.0000)	−2.276763^b^ (0.0114)

% level.^a^ Denotes significance at the 1

% level.^b^ Denotes significance at the 5

Like test results mentioned by Pedroni [43], the Panel ADF and Group ADF test results were the best, Panel V and Group Rho are the worst, while the rest are intermediate. When the test conclusions were inconsistent, we followed this order to determine the co-integration relationship. According to this criterion, the Panel ADF, Group ADF, Panel PP, and Group PP tests rejected the original assumptions that there was no co-integration relationship at the 1% significance level for Eastern China. The Panel ADF, Panel PP, and Group PP tests were significant at 1% significance level and the Group ADF test at the 5% significance level, so we rejected the original assumption that there was no co-integration relationship for Central China. The Panel ADF, Panel PP, and Group PP tests are significant at the 1% significance level and the Group ADF test at the 5% significance level, so we rejected the original hypothesis that there was no co-integration in Western China. This proved that the four variables, i.e., LIEC, LPTI, LSS, and LFTD, had co-integration relationships in the three regions.

#### 3.3. Estimation model

The co-integration test showed that there were co-integration relationships between the per-capita carbon emissions and the energy structure, energy efficiency, and economic development in each economic region. The long-term equilibrium model of the regional per-capita carbon emissions and its factors is as follows.
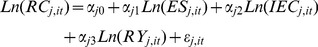
(7)where, j = 1, 2, 3, respectively, represent Eastern China, Central China, and Western China; i represents the cross-section of individuals; and t represents time. Thus, the regional estimation results were as follows.

Eastern China:
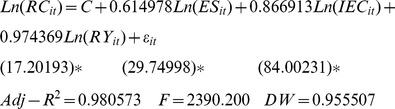
(8)


Central China:
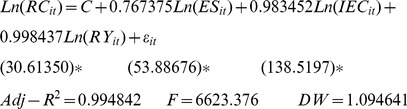
(9)


Western China:
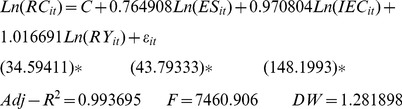
(10)


Initially, for the energy structure elasticity involved with regional per-capita carbon emissions: Eastern China = 0.615, Central China = 0.767, and Western China = 0.765. Thus, Central China ranked first, Western China second, and Eastern China third. After considering the energy efficiency elasticity, the results for Eastern China, Central China, and Western China were 0.867, 0.983, and 0.971 respectively, with the highest in Central China, the second in Western China, and the lowest in Eastern China. Finally, after considering the economic development elasticity, the results were 0.974 in Eastern China, 0.998 in Central China, and 1.017 in Western China, i.e., Western China, Central China, and Eastern China in descending order.

The energy structure is characterized as the coal proportion in the overall energy consumption and coal is the highest of all the carbon emission coefficients of primary energy, so the energy structure elasticities of the per-capita carbon emissions were positive in Eastern China, Central China, and Western China. As a result, the energy structures in Eastern China, Central China, and Western China were 68.0%, 89.5%, and 76.4%, respectively, in 1997, but 59.6%, 83.8%, and 76.5%, in 2009. The energy structure and energy structure elasticity of the per-capita carbon emissions shared identical sequences in Eastern China, Central China, and Western China so the higher coal reserves and production in Central China and Western China led to a higher coal proportion of the energy consumption and a rapid increase in carbon emissions and per-capita carbon emissions.

In conclusion, a higher energy intensity (energy consumption per unit GDP) was linked to higher per-capita carbon emissions, which agreed with our theoretical expectations. The energy intensity was 1.475, 2.044, and 2.439 in Eastern China, Central China, and Western China, respectively, as tons of standard coal per million yuan in 1997, which changed to be 1.149, 1.543, and 1.855 in 2009, i.e., decreases of 22.1%, 24.5%, 23.9%, respectively. The extent of the reduction kept pace with the energy efficiency elasticity of the per-capita carbon emissions in the three regions.

The per-capita GDPs in Eastern China, Central China, and Western China were 9,284 yuan, 4,957 yuan, and 3,883 yuan, respectively, in 1997, which soared to 28,678 yuan, 14,639 yuan, and 13,038 yuan in 2009, with an annual growth rate of the per-capita GDP from 1997 to 2009 of 9.9%, 9.4%, and 10.6%. In general, a higher economic development level led to higher individual consumption levels and more carbon emissions were generated. Western China’s per-capita GDP grew at the highest rate from 1997 to 2009. Due to the low level of economic development in Western China, its per-capita carbon emissions level ranked last among the three regions in 1997, whereas they exceeded those of Central China in 2009. Therefore, from 1997 to 2009, the economic development elasticity of the regional per-capita carbon emissions was highest in Western China but lowest in Eastern China, which experienced excessive per-capita carbon emissions accompanied by rapid economic development.

#### 3.4. The Short-term Factors that Affected the Regional Per-capita Carbon Emissions (Error Correction Model)

Traditionally, an econometric model is specified based on a particular economic theory or the recognition of economic behaviors, which help to clarify the theoretical relationship between the model variables. However, the co-integration and error correction model determined the variables and the relationships between them by referring to specific relationships derived from the data with respect to economic variables. We used the Engle-Granger two-step approach to establish the panel error correction model and to examine the short-term dynamics of changes in China’s per-capita carbon emissions. The model was as follows.
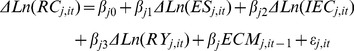
(11)


Variations in the regional per-capita carbon emissions can be divided into long-term equilibrium and short-term fluctuations. The error correction term in the model reflects the intensity of adjusting the per-capita carbon emissions that deviate from the long-terms equilibrium. Eq. (11) was used to estimate the regional error correction models, as shown in Table 4.

**Table 4:** Results of the regional error correction model.

Variable	Eastern China	Central China	Western China
Δ*Ln(ES)*	0.730269^a^ (0.0000)	0.788592^a^ (0.0000)	0.849344^a^ (0.0000)
Δ*Ln(IEC)*	0.830839^a^ (0.0000)	0.902072^a^ (0.0000)	0.903627^a^ (0.0000)
Δ*Ln(RY)*	0.762260^a^ (0.0000)	0.943951^a^ (0.0000)	0.955296^a^ (0.0000)
*ECM*(−1)	−0.032685 (0.2261)	−0.088058^b^ (0.0237)	−0.170012^a^ (0.0002)
*Adj-R^2^*	0.750556	0.924535	0.919809
*F*	99.54208	291.9669	376.6519
*DW*	2.708426	2.601132	2.387377

% level.^a^ Denotes significance at the 1

% level.^b^ Denotes significance at the 5

The error correction model demonstrates that in the short-term the effects of the regional energy structure, energy efficiency (energy intensity), and economic development on the per-capita carbon emissions Western China, Central China, and Eastern China ranked first, second, and third, respectively. In addition, each region’s error correction model (ECM) terms showed that the theoretical assumptions matched the reality. The ECM coefficients for Eastern China, Central China, and Western China were –0.0327, –0.0881, and –0.1700, respectively. Of these, Western China adjusted to the balanced state at the fastest rate, followed by Central China and Eastern China.

## Conclusion and Suggestions

The average annual growth of China’s per-capita carbon emissions was 7.1% between 1997 and 2009. In 1997, Eastern China, Central China, and Western China ranked first, second, and third in the per-capita carbon emissions, while in 2009 the ranking changed to Eastern China, Western China, and Central China, where Western China exceeded Central China. The LMDI decomposition results showed that economic growth stimulated the increase of per-capita carbon emissions in Eastern China, Central China, and Western China between 1997 and 2009. It is notable that the effects of economic growth on the per-capita carbon emissions in Eastern China were similar in Central China, but greater in Western China. In addition, the inhibitory effects of energy efficiency on the increased per-capita carbon emissions were much higher than those of the energy structure. Furthermore, the inhibitory effect of improved energy efficiency on the per-capita carbon emissions in Eastern China were similar to that in Central China, but larger than that in Western China. The inhibitory effect of the energy structure’s change on the increased per-capita carbon emissions was very weak, i.e., <1% in Eastern China, Central China, and Western China. The analysis of the factors that affected the regional per-capita carbon emissions using a co-integration test indicated that for the energy structure elasticity involved with the regional per-capita carbon emissions, Central China ranked first, followed by Western China and Eastern China. After considering the energy efficiency elasticity, Central China was highest, followed by Western China and Eastern China. Finally, when the economic development elasticity was considered, Western China was highest, followed by Central China and Eastern China.

The LMDI decomposition results showed that the continued growth of GDP per capita was the dominant factor that led to the growth of per-capita carbon emissions, while the stimulating effect of economic development on the carbon emissions per-capita in Central China was greater than that in Eastern China, and that in Eastern China was also greater than that in Western China. As a developing country, the growth of the economic development and GDP per-capita is necessary to meet the national needs and development requirements, and energy consumption is the basic input that maintains the normal operation of the economic system. Thus, an increased pressure on the environment is inevitable. China’s current stage of development means that the continued growth of China’s carbon emissions and per-capita carbon emissions are unavoidable in the future for a long period of time. Furthermore, according to the current international division of labor, China produces many high energy-consuming products for developed countries, which makes it more difficult for China to access the peak phase of carbon emissions than developed countries. The results of the LMDI decomposition and co-integration analysis showed that economic development was the most important factor that affects the growth of per- capita carbon emissions. However, economic growth will not lead to a reduction in carbon emissions immediately, and it need not lead to the growth of total carbon emissions. Thus, it is possible to slow the growth in the per- capita carbon emissions through government-led emission reduction measures, which can control the carbon emissions growth during the economic development process.

First, it is necessary to strengthen efforts to optimize the industrial structure and promote the development of low-carbon industries. Based on administrative and economic measures, governments can facilitate resource integration in the high energy-consuming heavy industry to limit the development of high-carbon industry. There should be an aim to develop tertiary industry because the energy intensity per GDP in the tertiary industry is less than one-quarter of that in the secondary industry. In 2012, China’s tertiary industry accounted for 44.6% of the total, whereas it comprised two-thirds of the total in developed countries, so the development of China’s tertiary industry still has great potential. There should be a focus on actively developing a modern service industry and high-tech industries, and using financial measures to support the development of the information industry, eco-industries, new energy development, and other green industries.

Second, there is a need to fundamentally change the high input, high energy consumption, high pollution, and low efficiency growth pattern, and adopt an intensive growth mode instead of an extensive one. The LMDI decomposition results showed that one-quarter of the per-capita carbon emissions growth caused by economic development was offset by an increase in the energy efficiency, so energy savings and energy efficiency improvement are the most effective ways to reduce emissions. According to a study of China’s Energy Development Strategy and Policy Research Group, the energy consumption per unit product in energy-intensive industries was 21% higher than that of the world’s advanced level, which shows that there is still great potential for China to slow the growth of per-capita carbon emissions by continuing to improve the energy efficiency and reducing the carbon emissions per unit of GDP in the future. China should actively encourage the development and application of low carbon technologies, transform the steel, cement, and other high-carbon industries using high-tech approaches, promote industrial innovation and upgrading within the industry, and focus on the promotion and application of advanced emission reduction technologies. China can also strengthen international cooperation, introduce advanced energy-saving technologies such as clean coal technology from developed countries and promote them, as well as encouraging the use of the Clean Development Mechanism (CDM), carbon capture, and storage technology (CSS) in related units. Moreover, China can strengthen energy management to improve energy efficiency in many ways. For example, relevant laws and regulations could be established and improved to promote the development of a low-carbon economy, by setting enforced carbon intensity standards for various industries and by encouraging families to adopt low-carbon lifestyles by economic incentives.

Third, there is a need to optimize the energy consumption structure. The decomposition results showed that the energy structure could limitedly slow the growth of per-capita carbon emissions, but this is because the coal-dominated energy consumption structure has not changed significantly in the last decade. China’s coal-based energy consumption structure means that it is difficult to change rapidly. However, it is China’s long-term aim to improve the energy structure by actively increasing oil and gas imports, developing new energy and renewable energy, and gradually reducing the weighting of coal in the overall energy consumption. China has abundant hydropower, wind, and solar energy, but the main factors that restrict renewable energy development at present are the cost and technology. Therefore, governments need to respond by supporting new energy through loans, taxes, and other measures. The growth of per-capita carbon emissions can be slowed by the establishment of a new “low carbon” or even “zero carbon” energy system by developing and utilizing new energy and renewable energy sources.

Finally, our results show that the current status and the trends in the per-capita carbon emissions in each region varied dramatically. Thus, efforts to narrow the gap in regional per-capita carbon emissions will help to lower their growth rate. To achieve the energy-saving objectives of China the circulation barriers should be eliminated, and low-carbon technologies could be shared within regions and resources to flow freely, thereby ensuring their optimal allocation.

## Supporting Information

Appendix S1 **Supporting tables.** Table S1, The LMDI-based decomposition results (1997–1998). Table S2, The LMDI-based decomposition results (1998–1999). Table S3, The LMDI-based decomposition results (1999–2000). Table S4, The LMDI-based decomposition results (2000–2001). Table S5, The LMDI-based decomposition results (2001–2002). Table S6, The LMDI-based decomposition results (2002–2003). Table S7, The LMDI-based decomposition results (2003–2004). Table S8, The LMDI-based decomposition results (2004–2005). Table S9, The LMDI-based decomposition results (2005–2006). Table S10, The LMDI-based decomposition results (2006–2007). Table S11, The LMDI-based decomposition results (2007–2008). Table S12, The LMDI-based decomposition results (2008–2009).
(DOC)

## References

[1] Mielnik O, Goldemberg J (1999) The evolution of the “carbonization index” in developing countries. Energy Policy 27: 307–308.

[2] Ang BW (1999) Is the Energy intensity a less useful indicator than the carbon factor in the study of climate change. Energy Policy 27: 943–946.

[3] Zhang ZQ, Qu JS, Zeng JJ (2008) A quantitative comparison and analysis on the assessment indicators of greenhouse gases emission. Journal of Geographical Sciences 18: 397–399.

[4] Sun JW (2005) The decrease of CO_2_ emission intensity is decarbonization at national and global levels. Energy Policy 33: 975–978.

[5] Lin BQ, Li XH (2011) The effect of carbon tax on per capita CO_2_ emissions. Energy Policy 39: 5137–5146.

[6] Jorgenson AK, Clark B (2013) The relationship between national-level carbon dioxide emissions and population size: an assessment of regional and temporal variation, 1960–2005. PloS one 8: e57107. 23437323 10.1371/journal.pone.0057107 PMC3577780

[7] Lanne M, Liski M (2004) Trends and breaks in per-capita carbon dioxide emissions, 1870–2028. Energy Journal 25: 41–65.

[8] Wang QQ, Huang XJ, Chen ZG, Tan D, Chuai XW (2009) Movement of the gravity of carbon emissions per capita and analysis of causes. Journal of Natural Resources 24: 833–841 (In Chinese with English abstract.)..

[9] Tian LX, Zhang BB (2011) Factor decomposition analysis of carbon emissions change in China. China Population, Resources and Environment 21: 1–7 (In Chinese with English abstract.)..

[10] Rajaratnam S, Kanthi P (2010) Is there a cointegrating relationship between Australia’s fossil-fuel based carbon dioxide emissions per capita and her GDP per capita?. Journal International Journal of Oil, Gas and Coal Technology 3: 1753–3309.

[11] Lee CC, Chang CP (2008) New evidence on the convergence of per capita carbon dioxide emissions from panel seemingly unrelated regressions augmented Dickey–Fuller tests. Energy 33: 1468–1475.

[12] Zaim O, Taskin F (2000) Environmental efficiency in carbon dioxide emissions in the OECD: a non-parametric approach. Journal of Environmental Management 58: 95–107.

[13] Zofío JL, Prieto AM (2001) Environmental efficiency and regulatory standards: the case of CO_2_ emissions from OECD industries. Resource and Energy Economics 23: 63–83.

[14] Zhou P, Ang BW, Poh KL (2006) Slacks-based efficiency measures for modeling environmental performance. Ecological Economics 60: 111–118.

[15] Lozano S, Gutiérrez E (2008) Non-parametric frontier approach to modelling the relationships among population, GDP, energy consumption and CO_2_ emissions. Ecological Economics 66: 687–699.

[16] Zhou P, Ang BW, Han JY (2010) Total factor carbon emission performance: a Malmquist index analysis. Energy Economics 32: 194–291.

[17] Wang QW, Zhou P, Zhou DQ (2012) Efficiency measurement with carbon dioxide emissions: the case of China. Applied Energy 90: 161–166.

[18] Fan Y, Liu LC, Wu G, Tsai HT, Wei YM (2007) Changes in carbon intensity in China: Empirical findings from 1980–2003. Ecological Economics 62: 683–691.

[19] Sun JW (1998) Accounting for energy use in China, 1980–94. Energy 23: 835–849.

[20] Zhang M, Mu HL, Ning YD (2009) Accounting for energy-related CO_2_ emission In China, 1991–2006. Energy Policy 37: 767–773.

[21] Ang BW, Zhang FQ, Choi KH (1998) Factorizing changes in energy and environmental indicators through decomposition. Energy 23: 489–495.

[22] Chunbo M, David IS (2008) China’s changing energy intensity trend: A decomposition analysis. Energy Economics 30: 1037–1053.

[23] Tunç GI, Serap TA, Elif A (2009) A decomposition analysis of CO_2_ emissions from energy use: Turkish case. Energy Policy 37: 4689–4699.

[24] Claudia S, Leticia O, Daniel C (2010) Using logarithmic mean Divisia index to analyze changes in energy use and carbon dioxide emissions in Mexico’ s iron and steel industry. Energy Economics 32: 1337–1344.

[25] Ang BW (2004) Decomposition analysis for policy making in energy: which is the preferred method?. Energy Policy 32: 1131–1139.

[26] Ang BW, Mu AR, Zhou P (2010) Accounting frameworks for tracking energy efficiency trends. Energy Economics 32: 1209–1219.

[27] Dong J, Wu M (2010) Energy performance index based on LMDI technique and decomposition analysis of Beijing’s energy consumption. Future Information Technology and Management Engineering (FITME), 2010 International Conference on IEEE 1: 246–249.

[28] Baležentis A, Baležentis T, Streimikiene D (2011) The energy intensity in Lithuania during 1995–2009: a LMDI approach. Energy Policy 39: 7322–7334.

[29] Su B, Ang BW (2012a) Structural decomposition analysis applied to energy and emissions: aggregation issues. Economic Systems Research 24: 299–317.

[30] Su B, Ang BW (2012b) Structural decomposition analysis applied to energy and emissions: some methodological developments. Energy Economics 34: 177–188.

[31] Su B, Huang HC, Ang BW, Zhou P (2010) Input–output analysis of CO_2_ emissions embodied in trade: the effects of sector aggregation. Energy Economics 32: 166–175.

[32] Su B, Ang BW (2010) Input–output analysis of CO_2_ emissions embodied in trade: the effects of spatial aggregation. Ecological Economics 70: 10–18.

[33] Wang Y, Zhao HY, Li LY, Liu Z, Liang S (2013) Carbon dioxide emission drivers for a typical metropolis using input–output structural decomposition analysis. Energy Policy 58: 312–318.

[34] Feng KS, Siu YL, Guan D, Hubacek K (2012) Analyzing drivers of regional carbon dioxide emissions for China. Journal of Industrial Ecology 16: 600–611.

[35] Xu M, Li R, Crittenden JC, Chen YS (2011) CO_2_ emissions embodied in China’s exports from 2002 to 2008: a structural decomposition analysis. Energy Policy 39: 7381–7388.

[36] Du HB, Guo JH, Mao GZ, Smith AM, Wang XX, et al (2011) CO_2_ emissions embodied in China–US trade: Input–output analysis based on the emergy/dollar ratio. Energy Policy 39: 5980–5987.

[37] CNBSa (China’s National Bureau of Statistics) (2000, 2003, 2005, 2006, 2007, 2008, 2009, 2010, 2011) China Energy Statistic Yearbook 1997–1999, 2000–2002, 2004, 2005, 2006, 2007, 2008, 2009, 2010. Beijing: China Statistical Press (In Chinese).

[38] Xu GQ, Liu ZY, Jiang ZH (2006) Decomposition model and empirical study of carbon emissions for China, 1995–2004. China Population, Resources and Environment 16: 158–61 (In Chinese with English abstract.)..

[39] Hu C, Huang XJ (2008) Characteristics of carbon emission in China and analysis on its cause. China Population, Resources and Environment 18: 38–42.

[40] IPCC (2006) IPCC guidelines for national greenhouse gas inventories: Volume II [EB/OL].

[41] CNBSb (China’s National Bureau of Statistics) (1999, 2000, 2001, 2002, 2003, 2004, 2005, 2006, 2007, 2008, 2009, 2010, 2011) China Statistic Yearbook 1998–2010. Beijing: China Statistical Press (In Chinese).

[42] Harris DF, Tzavalis E (1999) Inference for Unit Roots In Dynamic Panels Where The Time Dimension Is Fixed. Journal of Econometrics 91: 201–226.

[43] Pedroni P (1999) Critical Values for Cointegration Test In Heterogeneous Panels with Multiple Regressors. Oxford Bulletin of Economics and Statistics 61: 653–670.

