# Supplementary material for: Factors Affecting Regional Per-Capita Carbon Emissions in China Based on an LMDI Factor Decomposition Model
Source: PLoS One. 2013 Dec 6;8(12):e80888. doi: 10.1371/journal.pone.0080888 (PMC3865993; doi:10.1371/journal.pone.0080888)
Supplement: Appendix S1 — Supporting tables. Table S1, The LMDI-based decomposition results (1997–1998). Table S2, The LMDI-based decomposition results (1998–1999). Table S3, The LMDI-based decomposition results (1999–2000). Table S4, The LMDI-based decomposition results (2000–2001). Table S5, The LMDI-based decomposition results (2001–2002). Table S6, The LMDI-based decomposition results (2002–2003). Table S7, The LMDI-based decomposition results (2003–2004). Table S8, The LMDI-based decomposition results (2004–2005). Table S9, The LMDI-based decomposition results (2005–2006). Table S10, The LMDI-based decomposition results (2006–2007). Table S11, The LMDI-based decomposition results (2007–2008). Table S12, The LMDI-based decomposition results (2008–2009). (DOC) [file pone.0080888.s001.doc]

**Appendix S1 The decomposition results for each region and year**

**Table S1 The LMDI-based decomposition results (1997-1998)**

| Region | Share of the energy structure factor | Share of the energy efficiency factor | Share of the economic development factor | Region | Share of the energy structure factor | Share of the energy efficiency factor | Share of the economic development factor |
| --- | --- | --- | --- | --- | --- | --- | --- |
| Beijing | 0.0252 | -5.5801 | 6.5549 | Hunan | 0.0261 | -8.0407 | 9.0146 |
| Tianjin | -0.1055 | -10.3157 | 11.4212 | Guangdong | -0.0515 | -0.8426 | 1.8940 |
| Hebei | -0.0938 | -13.0019 | 12.0958 | Guangxi | -0.1647 | -2.0167 | 3.1815 |
| Shanxi | 0.0025 | -1.6149 | 2.6124 | Hainan | 0.0003 | 0.7931 | 0.2065 |
| Inner Mongolia | 0.0001 | -1.8430 | 0.8429 | Chongqing | 0.0073 | -0.3326 | 1.3253 |
| Liaoning | 0.0084 | -3.2660 | 2.2575 | Sichuan | -1.1481 | -28.7875 | 30.9357 |
| Jilin | -0.0319 | -1.5924 | 0.6243 | Guizhou | 0.0001 | -0.0968 | 1.0967 |
| Heilongjiang | -0.0247 | -1.5190 | 0.5437 | Yunnan | -0.0070 | -2.8411 | 1.8481 |
| Shanghai | -0.1555 | -10.3645 | 11.5200 | Shaanxi | -0.0581 | -3.3863 | 2.4444 |
| Jiangsu | -0.0301 | -7.4292 | 8.4593 | Gansu | -0.1075 | -2.3671 | 3.4746 |
| Zhejiang | -0.0211 | -6.4321 | 5.4532 | Qinghai | -0.1278 | -6.2640 | 5.3918 |
| Anhui | -0.0050 | 0.0989 | 0.9061 | Ningxia | -1.0269 | -71.7666 | 71.7935 |
| Fujian | 0.0143 | -2.5006 | 3.4863 | Xinjiang | -0.2162 | -10.7466 | 11.9628 |
| Jiangxi | -0.0651 | -3.2904 | 2.3555 | Eastern China | -0.6794 | -119.8865 | 119.5659 |
| Shandong | 0.0509 | -6.1876 | 5.1367 | Central China | -0.0302 | -6.3779 | 5.4081 |
| Henan | -0.0352 | -5.2023 | 6.2375 | Western China | -0.2208 | -13.6215 | 12.8423 |
| Hubei | 0.0440 | -5.4862 | 4.4421 | China | -0.1091 | -14.0859 | 13.1950 |

**Table S2 The LMDI-based decomposition results (1998-1999)**

| Region | Share of the energy structure factor | Share of the energy efficiency factor | Share of the economic development factor | Region | Share of the energy structure factor | Share of the energy efficiency factor | Share of the economic development factor |
| --- | --- | --- | --- | --- | --- | --- | --- |
| Beijing | -0.2844 | -4.2347 | 5.5192 | Hunan | -0.0270 | -1.1663 | 0.1933 |
| Tianjin | -0.1458 | -2.8624 | 4.0082 | Guangdong | -0.0242 | -0.6792 | 1.7034 |
| Hebei | 0.0237 | -6.4385 | 7.4148 | Guangxi | -0.1606 | -5.5897 | 6.7503 |
| Shanxi | 0.0028 | 0.4826 | 0.5147 | Hainan | 0.0462 | -1.5292 | 0.4830 |
| Inner Mongolia | 0.0061 | -0.8876 | 1.8816 | Chongqing | -0.0413 | 0.5112 | 0.5302 |
| Liaoning | -0.6257 | -14.2550 | 15.8807 | Sichuan | -0.0567 | -1.2440 | 0.3006 |
| Jilin | 0.1245 | -3.4190 | 2.2945 | Guizhou | -0.0158 | -1.7933 | 0.8091 |
| Heilongjiang | -0.1803 | -2.7026 | 1.8828 | Yunnan | -0.0253 | -1.5228 | 0.5481 |
| Shanghai | -0.0837 | -2.9940 | 4.0777 | Shaanxi | -0.0319 | -1.5893 | 0.6212 |
| Jiangsu | 0.3803 | -30.5964 | 31.2162 | Gansu | 0.1791 | -7.5163 | 6.3372 |
| Zhejiang | -0.0449 | -0.8133 | 1.8583 | Qinghai | 0.0009 | 0.4630 | 0.5361 |
| Anhui | 0.0112 | -1.9279 | 2.9167 | Ningxia | -0.0547 | -2.8234 | 1.8780 |
| Fujian | 0.0009 | 0.2692 | 0.7299 | Xinjiang | -0.1925 | -0.8758 | 2.0683 |
| Jiangxi | -0.0914 | 0.9053 | 0.1860 | Eastern China | -0.0650 | -3.7421 | 4.8071 |
| Shandong | 0.2567 | -50.7936 | 49.5370 | Central China | -0.0279 | -1.7976 | 0.8255 |
| Henan | -0.0122 | -1.4506 | 2.4628 | Western China | -0.0682 | -2.2251 | 1.2933 |
| Hubei | -0.0558 | -1.3472 | 2.4030 | China | -0.0999 | -4.6959 | 3.7958 |

**Table S3 The LMDI-based decomposition results (1999-2000)**

| Region | Share of the energy structure factor | Share of the energy efficiency factor | Share of the economic development factor | Region | Share of the energy structure factor | Share of the energy efficiency factor | Share of the economic development factor |
| --- | --- | --- | --- | --- | --- | --- | --- |
| Beijing | -0.0602 | -1.7862 | 0.8464 | Hunan | -0.0588 | -2.4331 | 1.4919 |
| Tianjin | -0.0502 | -0.0285 | 1.0787 | Guangdong | 0.9057 | -16.3411 | 16.4353 |
| Hebei | -0.0194 | -1.1730 | 2.1925 | Guangxi | -0.0344 | 0.7315 | 0.3029 |
| Shanxi | 0.0032 | -1.4488 | 2.4456 | Hainan | -0.0282 | 0.1572 | 0.8710 |
| Inner Mongolia | -0.0076 | -0.0558 | 1.0635 | Chongqing | -0.8924 | -6.7270 | 6.6195 |
| Liaoning | -0.0184 | 0.4275 | 0.5909 | Sichuan | -0.2335 | -3.2799 | 4.5134 |
| Jilin | -0.1603 | -10.7700 | 9.9303 | Guizhou | -0.0185 | -2.8870 | 3.9055 |
| Heilongjiang | -0.0564 | -0.8720 | 1.9284 | Yunnan | -0.0206 | 0.2304 | 0.7902 |
| Shanghai | 0.1666 | 0.3953 | 0.4381 | Shaanxi | -2.2490 | -18.3966 | 19.6457 |
| Jiangsu | -0.1170 | -0.9457 | 2.0627 | Gansu | -0.0605 | 0.7112 | 0.3493 |
| Zhejiang | -0.0470 | -0.0279 | 1.0749 | Qinghai | -0.0588 | -1.3881 | 0.4470 |
| Anhui | 0.0035 | 0.7338 | 0.2627 | Ningxia | -0.1828 | -1.0779 | 2.2607 |
| Fujian | -0.0014 | 0.0945 | 0.9068 | Xinjiang | 0.0552 | -2.8729 | 1.8177 |
| Jiangxi | 0.0040 | -1.4100 | 2.4060 | Eastern China | -0.0805 | -1.3943 | 2.4748 |
| Shandong | -0.0461 | -1.7090 | 0.7552 | Central China | -0.0264 | -1.7666 | 2.7930 |
| Henan | -0.0080 | -1.0846 | 2.0925 | Western China | -0.1074 | -1.3904 | 2.4978 |
| Hubei | 0.0148 | -15.0043 | 15.9895 | China | -0.0664 | -1.5421 | 2.6085 |

**Table S4 The LMDI-based decomposition results (2000-2001)**

| Region | Share of the energy structure factor | Share of the energy efficiency factor | Share of the economic development factor | Region | Share of the energy structure factor | Share of the energy efficiency factor | Share of the economic development factor |
| --- | --- | --- | --- | --- | --- | --- | --- |
| Beijing | -0.5304 | -13.4598 | 12.9902 | Hunan | 0.0358 | 0.6219 | 0.3423 |
| Tianjin | 0.0144 | -1.1439 | 2.1295 | Guangdong | -0.0316 | -0.3683 | 1.3999 |
| Hebei | 0.0313 | -1.1841 | 2.1527 | Guangxi | -0.1201 | -0.7776 | 1.8977 |
| Shanxi | -0.0100 | -0.4165 | 1.4264 | Hainan | -0.0329 | 0.7807 | 0.2522 |
| Inner Mongolia | -0.0075 | -0.2499 | 1.2574 | Chongqing | 0.0551 | -1.4551 | 0.4000 |
| Liaoning | -0.9203 | -7.7649 | 9.6852 | Sichuan | -0.0695 | -3.5085 | 2.5780 |
| Jilin | 0.0241 | -0.8047 | 1.7806 | Guizhou | -0.0229 | -2.3188 | 1.3417 |
| Heilongjiang | 0.0139 | -2.2696 | 1.2557 | Yunnan | -0.0899 | 0.4899 | 0.6000 |
| Shanghai | -0.0212 | -0.4273 | 1.4485 | Shaanxi | -0.0202 | 0.4568 | 0.5634 |
| Jiangsu | 0.0856 | -7.5446 | 8.4590 | Gansu | 0.1398 | -2.9067 | 1.7669 |
| Zhejiang | 0.0014 | -0.0315 | 1.0301 | Qinghai | 0.0026 | 0.4201 | 0.5773 |
| Anhui | 0.0129 | 0.1149 | 0.8722 | Ningxia | 0.0115 | 0.8501 | 0.1384 |
| Fujian | 0.0097 | -3.8833 | 4.8736 | Xinjiang | -0.2358 | -1.0183 | 2.2541 |
| Jiangxi | 0.0133 | -0.6621 | 1.6488 | Eastern China | -0.0040 | -0.3441 | 1.3480 |
| Shandong | 0.0239 | 0.5855 | 0.3907 | Central China | 0.0367 | -0.8926 | 1.8560 |
| Henan | 0.0015 | -0.1269 | 1.1254 | Western China | 0.0083 | -1.0925 | 2.0842 |
| Hubei | 0.1351 | -5.8057 | 4.6706 | China | 0.0069 | -0.6268 | 1.6199 |

**Table S5 The LMDI-based decomposition results (2001-2002)**

| Region | Share of the energy structure factor | Share of the energy efficiency factor | Share of the economic development factor | Region | Share of the energy structure factor | Share of the energy efficiency factor | Share of the economic development factor |
| --- | --- | --- | --- | --- | --- | --- | --- |
| Beijing | -0.4685 | -3.2845 | 4.7530 | Hunan | -0.0601 | 0.3671 | 0.6930 |
| Tianjin | -0.0322 | -4.7461 | 3.7784 | Guangdong | 0.0022 | -0.1126 | 1.1104 |
| Hebei | 0.0073 | -0.1328 | 1.1255 | Guangxi | -1.1029 | -21.4550 | 21.5579 |
| Shanxi | 0.0020 | 0.3898 | 0.6082 | Hainan | -0.0533 | 0.7000 | 0.3533 |
| Inner Mongolia | 0.0027 | -0.2299 | 1.2272 | Chongqing | 0.0103 | -0.0968 | 1.0865 |
| Liaoning | 0.1948 | -4.4968 | 3.3020 | Sichuan | 0.0169 | 0.3452 | 0.6379 |
| Jilin | -0.0127 | -1.1370 | 2.1497 | Guizhou | 0.0118 | -0.9159 | 1.9041 |
| Heilongjiang | 0.0483 | -21.0141 | 21.9658 | Yunnan | -0.0061 | 0.2461 | 0.7601 |
| Shanghai | -0.0659 | -0.2497 | 1.3156 | Shaanxi | -0.0204 | 0.2229 | 0.7975 |
| Jiangsu | 0.0055 | -0.5338 | 1.5283 | Gansu | 0.1669 | -1.5518 | 2.3849 |
| Zhejiang | 0.0099 | -0.8902 | 1.8803 | Qinghai | -0.3742 | -0.9225 | 2.2966 |
| Anhui | -0.0006 | -0.5801 | 1.5807 | Ningxia | 0.0015 | 0.6079 | 0.3906 |
| Fujian | 0.0011 | 0.5792 | 0.4197 | Xinjiang | 0.1511 | -2.2558 | 3.1047 |
| Jiangxi | -0.1323 | -1.0388 | 2.1712 | Eastern China | -0.0012 | -0.3176 | 1.3188 |
| Shandong | -0.0044 | 0.3636 | 0.6408 | Central China | -0.0010 | 0.1404 | 0.8606 |
| Henan | 0.0131 | 0.0802 | 0.9068 | Western China | 0.0027 | -0.0319 | 1.0292 |
| Hubei | -0.0170 | 0.2262 | 0.7909 | China | 0.0004 | -0.1088 | 1.1084 |

**Table S6 The LMDI-based decomposition results (2002-2003)**

| Region | Share of the energy structure factor | Share of the energy efficiency factor | Share of the economic development factor | Region | Share of the energy structure factor | Share of the energy efficiency factor | Share of the economic development factor |
| --- | --- | --- | --- | --- | --- | --- | --- |
| Beijing | 0.3236 | -46.6027 | 45.2791 | Hunan | 0.0688 | 0.4572 | 0.4740 |
| Tianjin | 0.0253 | -0.1341 | 1.1088 | Guangdong | 0.0177 | -1.0646 | 2.0468 |
| Hebei | -0.0010 | -0.2494 | 1.2505 | Guangxi | 0.0218 | 0.3170 | 0.6612 |
| Shanxi | 0.0009 | -1.6355 | 2.6346 | Hainan | -0.0287 | 0.6918 | 0.3369 |
| Inner Mongolia | 0.0016 | -0.1026 | 1.1010 | Chongqing | -0.2987 | -3.4198 | 2.7185 |
| Liaoning | 0.0343 | 0.1307 | 0.8351 | Sichuan | 0.0277 | 0.7143 | 0.2580 |
| Jilin | -0.0079 | -0.2004 | 1.2083 | Guizhou | 0.0057 | 0.3882 | 0.6061 |
| Heilongjiang | 0.0235 | 0.8323 | 0.1442 | Yunnan | 0.0117 | 0.6320 | 0.3562 |
| Shanghai | -0.0419 | -2.4691 | 3.5110 | Shaanxi | 0.0041 | -0.7661 | 1.7620 |
| Jiangsu | -0.0073 | -0.0369 | 1.0442 | Gansu | -0.0369 | 0.2449 | 0.7920 |
| Zhejiang | 0.0066 | -0.1131 | 1.1065 | Qinghai | -0.0602 | 0.3545 | 0.7057 |
| Anhui | -0.0004 | 0.4427 | 0.5577 | Ningxia | -0.0353 | -0.2291 | 1.2644 |
| Fujian | 0.0066 | 0.8122 | 0.1812 | Xinjiang | -0.0229 | -0.2191 | 1.2420 |
| Jiangxi | -0.0009 | 0.4389 | 0.5620 | Eastern China | 0.0104 | -0.4950 | 1.4846 |
| Shandong | 0.0312 | 0.0425 | 0.9263 | Central China | 0.0049 | 0.4402 | 0.5549 |
| Henan | 0.0000 | 0.1869 | 0.8131 | Western China | 0.0015 | 0.3103 | 0.6882 |
| Hubei | 0.0110 | -1.6051 | 0.5941 | China | 0.0058 | -0.0297 | 1.0239 |

**Table S7 The LMDI-based decomposition results (2003-2004)**

| Region | Share of the energy structure factor | Share of the energy efficiency factor | Share of the economic development factor | Region | Share of the energy structure factor | Share of the energy efficiency factor | Share of the economic development factor |
| --- | --- | --- | --- | --- | --- | --- | --- |
| Beijing | 0.0128 | -1.3527 | 2.3399 | Hunan | -0.0032 | 0.3871 | 0.6161 |
| Tianjin | 0.0115 | -0.6552 | 1.6437 | Guangdong | -0.0107 | 0.1460 | 0.8647 |
| Hebei | 0.0064 | -0.0391 | 1.0327 | Guangxi | -0.0009 | 0.5011 | 0.4997 |
| Shanxi | -0.0031 | -0.7482 | 1.7513 | Hainan | 0.1306 | 0.2990 | 0.5705 |
| Inner Mongolia | -0.0109 | 0.3335 | 0.6774 | Chongqing | -0.0192 | 0.2587 | 0.7605 |
| Liaoning | -0.0013 | 0.7380 | 0.2633 | Sichuan | 0.0064 | 0.0536 | 0.9400 |
| Jilin | 0.0268 | -0.3035 | 1.2767 | Guizhou | 0.0050 | 0.4160 | 0.5789 |
| Heilongjiang | 0.0279 | 0.0402 | 0.9319 | Yunnan | 0.0715 | 0.0157 | 0.9127 |
| Shanghai | -0.0776 | -0.5258 | 1.6034 | Shaanxi | -0.0163 | 0.4578 | 0.5584 |
| Jiangsu | 0.0041 | 0.3733 | 0.6226 | Gansu | 0.2310 | -5.0885 | 5.8574 |
| Zhejiang | 0.0012 | 0.1732 | 0.8255 | Qinghai | -0.1789 | -1.7881 | 2.9670 |
| Anhui | 0.0017 | -2.3845 | 3.3827 | Ningxia | 0.0096 | -2.3809 | 1.3713 |
| Fujian | -0.0050 | 0.5333 | 0.4718 | Xinjiang | -0.0259 | 0.4653 | 0.5606 |
| Jiangxi | 0.0460 | 0.1229 | 0.8311 | Eastern China | 0.0019 | 0.2049 | 0.7932 |
| Shandong | 0.0008 | 0.1868 | 0.8124 | Central China | 0.0060 | 0.0458 | 0.9482 |
| Henan | -0.0005 | 0.4472 | 0.5534 | Western China | 0.0015 | 0.2261 | 0.7723 |
| Hubei | 0.0075 | -0.0357 | 1.0282 | China | 0.0028 | 0.1722 | 0.8250 |

**Table S8 The LMDI-based decomposition results (2004-2005)**

| Region | Share of the energy structure factor | Share of the energy efficiency factor | Share of the economic development factor | Region | Share of the energy structure factor | Share of the energy efficiency factor | Share of the economic development factor |
| --- | --- | --- | --- | --- | --- | --- | --- |
| Beijing | -0.0626 | 0.5459 | 0.5167 | Hunan | 0.0033 | 0.6035 | 0.3932 |
| Tianjin | 0.0119 | -1.1333 | 2.1214 | Guangdong | -0.0374 | 0.4406 | 0.5968 |
| Hebei | -0.0052 | 0.3208 | 0.6844 | Guangxi | -0.0113 | -0.0599 | 1.0712 |
| Shanxi | -0.0013 | 0.1388 | 0.8624 | Hainan | -0.0425 | -1.2545 | 0.2970 |
| Inner Mongolia | -0.0040 | -0.0045 | 1.0086 | Chongqing | 0.0007 | 0.1686 | 0.8306 |
| Liaoning | -0.0220 | 0.0051 | 1.0169 | Sichuan | -0.1047 | -2.5989 | 3.7035 |
| Jilin | -0.0196 | 0.5179 | 0.5017 | Guizhou | -0.0014 | -0.3639 | 1.3653 |
| Heilongjiang | 0.0124 | 0.1527 | 0.8348 | Yunnan | -0.0274 | 0.6851 | 0.3422 |
| Shanghai | -0.0746 | -0.4175 | 1.4922 | Shaanxi | 0.2147 | -1.0493 | 1.8346 |
| Jiangsu | 0.0053 | 0.2552 | 0.7396 | Gansu | -0.0266 | 0.1189 | 0.9077 |
| Zhejiang | -0.0009 | 0.3761 | 0.6248 | Qinghai | -0.1217 | -0.6759 | 1.7975 |
| Anhui | -0.0031 | -0.1373 | 1.1404 | Ningxia | 0.0774 | 0.1343 | 0.7883 |
| Fujian | 0.0112 | 0.5822 | 0.4066 | Xinjiang | -0.0374 | 0.2049 | 0.8325 |
| Jiangxi | -0.0303 | -0.1621 | 1.1924 | Eastern China | -0.0041 | 0.3801 | 0.6240 |
| Shandong | -0.0081 | 0.5352 | 0.4729 | Central China | -0.0025 | 0.2140 | 0.7885 |
| Henan | 0.0055 | 0.1041 | 0.8904 | Western China | 0.0008 | -0.0314 | 1.0306 |
| Hubei | -0.0238 | -0.0018 | 1.0256 | China | -0.0029 | 0.2257 | 0.7772 |

**Table S9 The LMDI-based decomposition results (2005-2006)**

| Region | Share of the energy structure factor | Share of the energy efficiency factor | Share of the economic development factor | Region | Share of the energy structure factor | Share of the energy efficiency factor | Share of the economic development factor |
| --- | --- | --- | --- | --- | --- | --- | --- |
| Beijing | -0.2467 | -1.7780 | 3.0247 | Hunan | -0.0079 | -0.3466 | 1.3545 |
| Tianjin | -0.0075 | -3.7853 | 2.7928 | Guangdong | 0.0046 | -0.3452 | 1.3407 |
| Hebei | 0.0060 | -1.5654 | 2.5594 | Guangxi | 0.0131 | -0.4003 | 1.3872 |
| Shanxi | -0.0033 | 0.1859 | 0.8174 | Hainan | -0.0541 | 0.2482 | 0.8058 |
| Inner Mongolia | -0.0096 | -0.1278 | 1.1374 | Chongqing | -0.0019 | 0.3040 | 0.6979 |
| Liaoning | 0.0008 | -0.4766 | 1.4757 | Sichuan | -0.0248 | -0.0024 | 1.0272 |
| Jilin | -0.0041 | -0.1659 | 1.1700 | Guizhou | 0.0018 | 0.2897 | 0.7085 |
| Heilongjiang | -0.0045 | -0.3891 | 1.3936 | Yunnan | 0.0034 | 0.1211 | 0.8754 |
| Shanghai | -0.1317 | 0.0605 | 1.0712 | Shaanxi | -0.0053 | 0.3020 | 0.7033 |
| Jiangsu | -0.0101 | -0.4968 | 1.5068 | Gansu | 0.0000 | -1.8405 | 2.8405 |
| Zhejiang | 0.0147 | 0.0472 | 0.9381 | Qinghai | 0.0552 | 0.3215 | 0.6233 |
| Anhui | -0.0136 | -0.4471 | 1.4606 | Ningxia | -0.0089 | -0.7994 | 1.8082 |
| Fujian | 0.0176 | -0.2341 | 1.2165 | Xinjiang | 0.0193 | -0.1408 | 1.1215 |
| Jiangxi | -0.0005 | -0.3595 | 1.3601 | Eastern China | -0.0043 | -0.3790 | 1.3833 |
| Shandong | 0.0117 | -0.1435 | 1.1319 | Central China | -0.0009 | -0.1044 | 1.1053 |
| Henan | 0.0043 | 0.0252 | 0.9705 | Western China | -0.0023 | 0.0377 | 0.9645 |
| Hubei | 0.0016 | -0.0540 | 1.0525 | China | -0.0024 | -0.1701 | 1.1726 |

**Table S10 The LMDI-based decomposition results (2006-2007)**

| Region | Share of the energy structure factor | Share of the energy efficiency factor | Share of the economic development factor | Region | Share of the energy structure factor | Share of the energy efficiency factor | Share of the economic development factor |
| --- | --- | --- | --- | --- | --- | --- | --- |
| Beijing | -0.6878 | -7.5666 | 9.2543 | Hunan | -0.0081 | -0.4279 | 1.4361 |
| Tianjin | -0.3229 | -6.0962 | 7.4191 | Guangdong | -0.0067 | 0.0136 | 0.9932 |
| Hebei | 0.0033 | 0.2402 | 0.7565 | Guangxi | 0.0087 | -0.1207 | 1.1120 |
| Shanxi | -0.0065 | -3.5820 | 4.5885 | Hainan | 0.0600 | 0.3575 | 0.5825 |
| Inner Mongolia | -0.0097 | -0.1061 | 1.1158 | Chongqing | -0.0057 | -0.0780 | 1.0837 |
| Liaoning | 0.0289 | -1.1455 | 2.1166 | Sichuan | 0.0059 | -0.1823 | 1.1765 |
| Jilin | -0.0368 | -1.2323 | 2.2691 | Guizhou | -0.0144 | -0.4735 | 1.4879 |
| Heilongjiang | 0.0169 | 0.1504 | 0.8327 | Yunnan | -0.0565 | -2.5272 | 3.5836 |
| Shanghai | -0.0706 | -1.8889 | 2.9595 | Shaanxi | -0.0479 | -0.2564 | 1.3043 |
| Jiangsu | -0.0110 | -0.2696 | 1.2806 | Gansu | 0.0093 | 0.0961 | 0.8946 |
| Zhejiang | 0.0150 | 0.1244 | 0.8606 | Qinghai | 0.1024 | -0.4454 | 1.3430 |
| Anhui | -0.0009 | -0.0896 | 1.0905 | Ningxia | 0.0085 | 0.0485 | 0.9431 |
| Fujian | 0.0020 | -0.0150 | 1.0130 | Xinjiang | 0.0280 | 0.1963 | 0.7758 |
| Jiangxi | 0.0197 | 0.0789 | 0.9014 | Eastern China | 0.0028 | -0.1255 | 1.1228 |
| Shandong | 0.0059 | -0.0547 | 1.0488 | Central China | -0.0055 | -0.5029 | 1.5084 |
| Henan | 0.0017 | -0.2376 | 1.2359 | Western China | -0.0040 | -0.1660 | 1.1700 |
| Hubei | -0.0182 | -0.1358 | 1.1539 | China | -0.0020 | -0.2118 | 1.2138 |

**Table S11 The LMDI-based decomposition results (2007-2008)**

| Region | Share of the energy structure factor | Share of the energy efficiency factor | Share of the economic development factor | Region | Share of the energy structure factor | Share of the energy efficiency factor | Share of the economic development factor |
| --- | --- | --- | --- | --- | --- | --- | --- |
| Beijing | -0.3113 | -0.7747 | 0.0859 | Hunan | 0.0180 | -6.1891 | 5.1711 |
| Tianjin | -0.4442 | -14.0011 | 13.4453 | Guangdong | 0.0335 | -1.1824 | 2.1489 |
| Hebei | -0.0836 | -11.0820 | 10.1656 | Guangxi | 0.0036 | -4.3078 | 3.3042 |
| Shanxi | -0.1767 | -9.2521 | 8.4288 | Hainan | -0.0018 | 0.3326 | 0.6692 |
| Inner Mongolia | 0.0009 | 0.0792 | 0.9200 | Chongqing | 0.0220 | 0.3703 | 0.6077 |
| Liaoning | -0.0767 | -5.4775 | 6.5542 | Sichuan | 0.0296 | 0.0792 | 0.8912 |
| Jilin | 5.9586 | -155.3109 | 148.3522 | Guizhou | -0.0223 | -2.3840 | 1.4063 |
| Heilongjiang | 0.0713 | -0.2664 | 1.1951 | Yunnan | -0.0126 | -1.7668 | 2.7794 |
| Shanghai | 0.1184 | -3.5498 | 4.4314 | Shaanxi | -0.0187 | -0.0038 | 1.0225 |
| Jiangsu | -0.0438 | -1.0914 | 2.1352 | Gansu | 0.0134 | -0.8797 | 1.8663 |
| Zhejiang | -0.5157 | -33.1868 | 32.7025 | Qinghai | 0.0058 | 0.4406 | 0.5536 |
| Anhui | 0.0038 | 0.2180 | 0.7782 | Ningxia | -0.0543 | -2.1987 | 3.2529 |
| Fujian | 0.0424 | -0.7185 | 1.6760 | Xinjiang | 0.1251 | -0.7164 | 1.5913 |
| Jiangxi | -0.0487 | -4.4391 | 5.4878 | Eastern China | -0.0234 | -1.8019 | 2.8252 |
| Shandong | 0.0119 | -0.5604 | 1.5485 | Central China | -0.0093 | -3.9797 | 4.9890 |
| Henan | -0.0262 | -3.5771 | 4.6033 | Western China | 0.0047 | -0.3002 | 1.2955 |
| Hubei | -2.1546 | -63.8638 | 67.0184 | China | -0.0065 | -1.2213 | 2.2279 |

**Table S12 The LMDI-based decomposition results (2008-2009)**

| Region | Share of the energy structure factor | Share of the energy efficiency factor | Share of the economic development factor | Region | Share of the energy structure factor | Share of the energy efficiency factor | Share of the economic development factor |
| --- | --- | --- | --- | --- | --- | --- | --- |
| Beijing | -0.1984 | -6.0809 | 5.2794 | Hunan | -0.0182 | -1.6045 | 2.6227 |
| Tianjin | -0.2567 | -32.1523 | 33.4090 | Guangdong | -0.1445 | -1.5940 | 2.7386 |
| Hebei | 0.0058 | 0.0974 | 0.8968 | Guangxi | 0.0030 | 0.1829 | 0.8141 |
| Shanxi | -0.0761 | -4.2696 | 3.3457 | Hainan | 0.1303 | -1.4877 | 2.3574 |
| Inner Mongolia | -0.0167 | -1.8251 | 2.8419 | Chongqing | 0.0299 | -2.5348 | 3.5050 |
| Liaoning | -0.0038 | -1.8208 | 2.8246 | Sichuan | -0.0066 | 0.0100 | 0.9966 |
| Jilin | -0.0226 | -3.9217 | 4.9443 | Guizhou | 0.0111 | -0.6018 | 1.5908 |
| Heilongjiang | -0.1215 | -0.1127 | 1.2342 | Yunnan | 0.0113 | 0.1992 | 0.7895 |
| Shanghai | -0.2878 | -9.7441 | 9.0319 | Shaanxi | 0.0365 | -2.9584 | 3.9219 |
| Jiangsu | -0.0587 | -5.2238 | 6.2825 | Gansu | -0.0911 | -3.6241 | 2.7152 |
| Zhejiang | -0.0725 | -1.0968 | 2.1693 | Qinghai | -0.0517 | -7.0207 | 6.0724 |
| Anhui | 0.0010 | -0.2646 | 1.2636 | Ningxia | 0.0098 | -1.2815 | 2.2718 |
| Fujian | -0.0217 | -0.6668 | 1.6885 | Xinjiang | 0.0804 | 0.8442 | 0.0754 |
| Jiangxi | 0.1481 | -44.1349 | 44.9867 | Eastern China | -0.0481 | -1.9706 | 3.0187 |
| Shandong | -0.0808 | -2.2049 | 3.2857 | Central China | -0.0310 | -2.4614 | 3.4924 |
| Henan | 0.0007 | -2.4938 | 3.4931 | Western China | 0.0104 | -0.6535 | 1.6431 |
| Hubei | 0.0085 | -0.8306 | 1.8221 | China | -0.0172 | -1.3769 | 2.3941 |
